# Evidence for parallel activation of the pre-supplementary motor area and inferior frontal cortex during response inhibition: a combined MEG and TMS study

**DOI:** 10.1098/rsos.171369

**Published:** 2018-02-14

**Authors:** Christopher Allen, Krish D. Singh, Frederick Verbruggen, Christopher D. Chambers

**Affiliations:** 1 Cardiff University Brain Research Imaging Centre, School of Psychology, Cardiff University, Maindy Road, Cardiff CF24 4HQ, UK; 2 Department of Experimental Psychology, Ghent University, Henri Dunantlaan 2, 9000 Ghent, Belgium; 3 Psychology, University of Exeter, Washington Singer Building, Perry Road, Exeter EX4 4QG, UK

**Keywords:** pre-supplementary motor area, inferior frontal cortex, magnetoencephalography, transcranial magnetic stimulation, timing, response inhibition

## Abstract

This pre-registered experiment sought to uncover the temporal relationship between the inferior frontal cortex (IFC) and the pre-supplementary motor area (pre-SMA) during stopping of an ongoing action. Both regions have previously been highlighted as being central to cognitive control of actions, particularly response inhibition. Here we tested which area is activated first during the stopping process using magnetoencephalography, before assessing the relative chronometry of each region using functionally localized transcranial magnetic stimulation. Both lines of evidence pointed towards simultaneous activity across both regions, suggesting that parallel, mutually interdependent processing may form the cortical basis of stopping. Additional exploratory analysis, however, provided weak evidence in support of previous suggestions that the pre-SMA may provide an ongoing drive of activity to the IFC.

## 1. Introduction

The process of stopping an ongoing action has become central to the study of cognitive control. The two cortical regions most often implicated in stopping are the inferior frontal cortex (IFC) and the pre-supplementary motor area (pre-SMA) (for review, see [1–3]). In this pre-registered study, we aimed to establish the temporal and causal interplay between these two regions. Few studies have addressed this question, but one earlier study, on a single subject, has used intracranial recording to demonstrate that activity related to stopping in the pre-SMA can precede IFC activation [4]. This suggests that when stopping an ongoing action, information may be transmitted from the pre-SMA to the right IFC, and that the pre-SMA may, therefore, be the cortical source of the ‘Stop’ signal. Recent modelling work has also suggested the primacy of the pre-SMA [5], although this may relate to an ongoing drive when preparing to stop, as opposed to the active response to stop an action [6].

**Figure 1.**
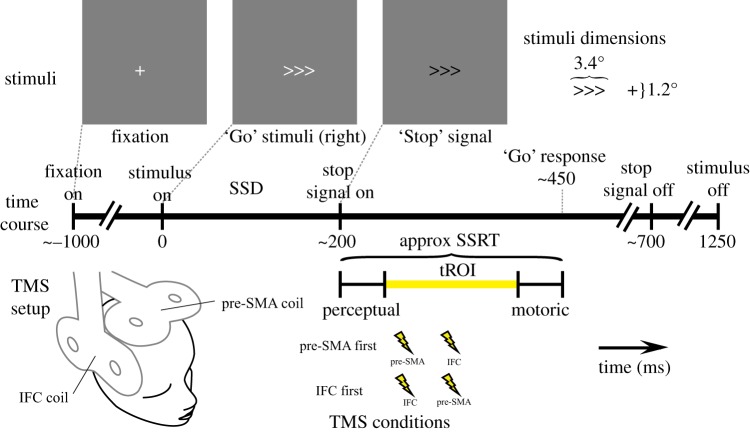
Illustration of the progression of a single trial where a ‘Stop’ signal is presented at 200 ms post stimulus onset. In this task, participants are asked to respond to the white arrow as quickly as possible with a directional button press, and to inhibit their response if a ‘Stop’ signal occurs (contrast reversal). Stimulus dimensions are indicated in degrees of visual angle. The delay between the onset of the ‘Go’ stimulus and the ‘Stop’ signal (the SSD) was dynamically altered to account for individual differences in performance. Also illustrated is the tROI where it is possible for regions under investigation to exert influence in response to the ‘Stop’ signal. TMS was applied to the pre-SMA and the IFC at 12.5% and 62.5% of each participant's tROI. An approximate stop signal reaction time (approx SSRT) is also shown for illustrative purposes.

Here we exploited the high temporal and reasonable spatial resolution of magnetoencephalography (MEG) and transcranial magnetic stimulation (TMS) to elucidate the temporal dynamics of the interplay between the IFC and the pre-SMA in a stop-signal task [7]. The time at which either cortical region is capable of exerting influence upon stopping an action is relatively limited. Previous research with similar paradigms has shown that the reaction time to a ‘Go’ signal is approximately 450 ms and the 50% stop signal delay (SSD) (the time between a ‘Go’ signal and a ‘Stop’ signal which results in successful stopping on 50% of trials) to be approximately 200 ms [8]. This leaves an interval of approximately 250 ms during which stopping must be implemented (figure 1). This period includes the time required for the sensory information to reach critical regions (approx. 50–100 ms [8,9]) and the duration of peripheral nerve transmission and muscle transduction when responding (approx. 30–50 ms [10,11]). If we further discount these phases, then a period of approximately 150 ms remains in which we can test the interplay between the IFC and the pre-SMA. This temporal region of interest (tROI) was the focus of this study, with the aim to uncover when and in what order these regions exert influence during the stopping of an action.

Using MEG, we examined four measures based on phenomena previously linked to the inhibition of actions; these included the evoked components [12–14], and three induced components divided according to frequency band: *α* [15–17], *β* [4,18–21] and *γ* [4,16]. The central question of the main analyses was which of the anatomical regions of interest (aROIs: IFG and pre-SMA) exhibited initial evidence of activity related to response inhibition. The data were also explored with Granger analysis [22,23].

In addition to MEG, TMS was applied to the same participants to test the causal direction of information transfer during stopping. The TMS was functionally guided according to MEG activations within both the IFC and pre-SMA. This functional localization resulted in IFC stimulation predominantly, but not exclusively, lateralized to the right IFC (15 out of 18 participants). Single pulses of TMS were administered using two coils to both the IFC and the pre-SMA, and the relative timing of TMS was alternated [24,25]. TMS was applied to both regions at either an earlier or later time point within each participant's functionally defined tROI (figure 1). The critical comparison was between two TMS conditions differentiated by the order in which TMS was applied, therefore, targeting the order of activity related to response inhibition between the two regions. If TMS applied to the pre-SMA before the IFC is more effective in disrupting participants' ability to stop than TMS applied initially to the IFC, then this would suggest that the flow of critical information proceeds in a pre-SMA-to-IFG direction, consistent with evidence reported by Swann *et al*. [4]. Conversely, if initial IFC stimulation were more effective, then this would imply temporal primacy of the IFC.

## 2. Material and methods

### 2.1. Overview

The protocol and analyses for this study were publically registered prior to data collection. The methods described here faithfully reflect those of the original registration document, unless otherwise stated (https://osf.io/rfxhn/). The two experimental components of this study (MEG and TMS) employed a common task. The experiments used the activations observed in the MEG experiment to determine which of the lateralized IFC sites was functionally relevant. TMS parameters, therefore, made use of information obtained from the MEG data, with the TMS session following the MEG session, both of which were preceded by an initial familiarization and calibration session. In the following sections, the task is described along with calibration procedures (§2.2). MEG experimental methods are then described (§2.3) followed by TMS methods (§2.4). A common statistical approach was used for the primary assessment of both experimental sections, described in §2.5. Exclusion criteria and participant instructions can be found in §§2.6 and 2.7. The data and many of the materials used have been made publically available at https://osf.io/qj66a/ and a document summarizing the content of the repository is available at https://osf.io/6s3kz/.

### 2.2. Behavioural task

#### 2.2.1. The task

The same basic behavioural task was used in both MEG and TMS sessions, based upon the classic stop-signal paradigm [7]. Each trial commenced with a fixation stimulus. In the calibration and TMS phases, the duration of the fixation interval was randomly jittered between the following values: 1000, 1200, 1400 ms; in the MEG phase, the duration of the fixation period was set at 1000 ms. The ‘Go’ stimulus, in this case a set of three arrows, was then presented and remained on screen for 1250 ms. Participants had to respond as quickly as possible to these arrows with a directional button press, using the index and middle finger of their preferred hand (figure 1). On a random one-third of trials, the arrow stimulus turned black after a variable delay, which was the signal to stop the response. The black arrows appeared for 500 ms. When the stop signal is presented rapidly after the ‘Go’ stimulus, participants find it relatively easy to stop. Conversely, at greater delays between ‘Go’ and ‘Stop’ signals it becomes harder to stop as the go process is closer to completion. Therefore, by adjusting the interval between ‘Go’ and ‘Stop’ signals (the SSD) it is possible to titrate participants' stopping performance, ensuring that stop and go processes are placed in optimal competition (50% SSD [26–28]). This involved a calibration phase in which we increased the SSD by a brief duration (50 ms, 3 screen refreshes at a 60 Hz vertical refresh rate) when participants successfully stopped a go response, and decreasing the SSD by the same amount when they failed to stop. Over a number of trials the SSD duration was extended and reduced, centring these reversals over each participant's specific SSD, which resulted in 50% successful stopping.

As illustrated in figure 1, the period during which either the pre-SMA or the IFC can exert influence over action is limited, being situated between onset of the ‘Stop’ signal and the action. The period of potential influence, or tROI is further theoretically restricted when considering that perceptual information must reach the aROIs prior to the period when they may exert influence, and that the regions of interest cannot be expected to exert influence during transmission of the motor responses in the peripheral nervous system. Here we made the relatively liberal assumption that both these perceptual and motoric periods must last at least 50 ms [9–11,29]. This resulted in a participant-specific tROI that was the target of both the MEG analyses and the TMS intervention.

#### 2.2.2. Procedure

Participants were familiarized with the task and parameters were calibrated during a session prior to MEG and TMS data acquisition. Initially participants were screened for suitability and asked to read the task instructions (see §2.7). They then completed a version of the task in which the SSD was dynamically altered to converge upon 50% successful stopping. Participants first completed 1–4 ‘mini’ blocks of 24 trials and then a number of larger blocks of 120 trials. Additional larger blocks were completed until the following criterion was fulfilled: (i) mean reaction time on ‘Go’ trials was less than 550 ms and (ii) the resulting SSD was greater than 100 ms. The initial SSD for all participants was set to 250 ms. All subsequent blocks took the resulting SSD from previous blocks as their starting points. If the mean reaction time on go trials was greater than 550 ms, participants were told to ‘Speed up your responses’ and if SSD was less than 100 ms they were told to ‘Try harder to stop’. If after 6 blocks of 120 trials they were unable to perform the task within these pre-registered bounds, they were excluded from the experiment (see §2.6).

The mean SSD within a block was then to be used to estimate the stop-signal reaction time (SSRT) using the integration method, which involves ordering ‘Go’ reaction times (RTs) in the block and establishing the RT that corresponds to the overall probability of responding on a probability distribution representative of all Go RTs within the block, and then subtracting the mean SSD from this derived RT [30,31].

During the calibration session, TMS intensity thresholds were also collected (see §2.4.2). In a separate session participants' T1-weighted anatomical MRI scans were obtained on a 3 T General Electric Signa-HDx system using an FSPGR sequence with the following parameters: 1 mm^3^ isotropic voxel size, field of view 256 × 192 × 176 mm or 256 × 256 × 256 mm (depending on head shape), TR: 7.9 ms, TE: 3.0 ms, TI 450 ms, flip angle 20^o^.

#### 2.2.3. Equipment

In the calibration and TMS sessions, stimuli were presented on a Mitsubishi Diamond Pro 2070sb CRT monitor with a 60 Hz refresh rate. The task was programmed in Matlab (MathWorks, UK) using the Psychophysics toolbox (v. 3.0.8). Responses were collected via a standard computer keyboard (see §2.7). Participants were seated in a semi-dark room facing the monitor with their position maintained by means of a chin and forehead rest. This presentation apparatus was also used in the TMS experiment.

#### 2.2.4. Participants

Twenty participants (14 female; age: mean = 24.9, s.d. = 5.6, range = 20 to 38 years) took part in the MEG and TMS experiments. The number of participants was based on the outcome of a Bayesian difference test [32] applied to the main comparisons (see §2.5.1). One participant was excluded from the MEG analyses on the basis of excessive stopping performance (proportion stopped during MEG blocks was 0.7, when the maximum permissible was 0.6; see pre-registered exclusion criterion in §2.6). None were excluded on the basis of pre-registered criteria for data quality. Of the 20 participants in the full experiment, 18 completed the TMS component (13 female; age: mean = 24.8, s.d. = 5.9, range = 20 to 38 years), due to 2 participants withdrawing, one of whom had a mild adverse reaction to the TMS. One participant was excluded from the TMS analysis, again due to excessive stopping performance. All data exclusions conformed to pre-registered rules.

### 2.3. Magnetoencephalography

#### 2.3.1. Magnetoencephalography overview

Our aim was to acquire activity traces, during the tROI, from each of the two aROIs and to then compare the latency at which either produced a detectable response. We first describe how the MEG data were acquired before outlining how information within the two aROIs was isolated (§§2.3.2–2.3.6). As the primary inferential statistics used to interpret the dependent measures are common to both TMS and MEG experimental sections, they are detailed in the combined statistics in §2.5.

#### 2.3.2. Procedure and acquisition parameters

Following familiarization and calibration, prior to MEG data collection, participants completed a behavioural block of 120 trials inside the MEG magnetically shielded room and the same criteria as used for the final block of the behavioural calibration phase were applied. This block was designed as a final calibration of the stimuli, to accommodate day-to-day variance in individual levels of performance.

The final SSD of the calibration block, theoretically corresponding to a 50% stopping rate, was then used for three subsequent blocks of 120 trials where MEG data were collected. The reason for using a constant duration, rather than adjusting the SSD dynamically, and for using a consistent fixation period during data collection, was to improve data quality by maintaining a consistent temporal structure between trials. To reduce the impact of variation in performance, participants were given feedback during the two inter-block intervals as follows: if participants' stopping rate exceeded 60%, they were told to ‘Remember to respond to the arrow as quickly as possible’, and if their stopping rate fell below 40%, they were told ‘Remember to try your best to stop’. This, together with the variable SSD encountered during training, discouraged participants from adopting a strategy involving waiting, but see also the exclusion criteria described in §2.6.

Stimuli were presented using a 60 Hz projector system (Sanyo, Pro-xtraX) targeted at a semi-opaque screen which was positioned above the participants while they lay in a supine position. To improve comfort, there was some flexibility in the screen positioning, resulting in approximation of the viewing angles previously described (figure 1). As with the calibration phase of the experiment, presentation was programmed using Matlab in conjunction with a Psychophysics toolbox, but here responses were collected via a Lumitouch (Photon Control Inc.) MEG-compatible response box.

MEG data were acquired on a 275-channel radial gradiometer system (CTF MEG, MEG International Services Ltd) sampled at 1200 Hz, with a 300 Hz anti-alias low-pass filter and analysed as third-order synthetic gradiometers [33]. Trials were divided into 2.6 s epochs, from 1.3 s before the onset of the first arrow stimuli (covering the fixation period) to 1.3 s after the first arrow's presentation (covering the response period; figure 1). Individual trial data were visually inspected for clearly corrupted sections (e.g. from movement) and the corresponding trials were excluded from subsequent MEG analyses. The experimenter was blinded to the stimulus condition during this procedure, which resulted in the exclusion of 3% of trials (216 of 7200 trials).

Participants completed the experiment in a supine posture, with head padding used to minimize head movement, and were instructed to maintain their head position to the best of their ability across all three acquisition blocks. Head localization was carried out at the start of each block, with the first position used for synthetic aperture magnetometry source localization (see §§2.3.4 and 2.3.5).

#### 2.3.3. Measures

The overall structure of the analysis was to first quantify brain activity over time within the two regions of interest, and to then compare their time courses. We considered four brain responses (classic evoked, *α*, *β*, *γ*), resulting in four dependent measures in the MEG analysis.

A common approach was applied to produce all four activity traces*,* where datasets were derived based upon a contrast between activity during ‘Stop’ trials and trials in which the participant was not instructed to stop, i.e. ‘Go’ trials. A secondary baseline consisting of the final 500 ms of the fixation period was also subtracted to produce an activity trace representing each participant's response to the stimuli. The structure of the contrast used to produce measures of interest can, therefore, be summarized as follows: (Stop–Go)–Fixation. The use of the baseline involving ‘Go’ trials was intended to remove activity associated with performing the motor ‘Go’ response, as well as the perceptual aspects due to the presentation of the arrow signal. Motor responses in particular have the potential to obscure activity drawn from the adjacent pre-SMA [34]. ‘Go’ trials were randomly down-sampled so that the numbers of ‘Go’ trials contributing to the MEG measures was equal to the number of ‘Stop’ trials. Datasets based upon these contrasts were used for all pre-specified analyses detailed here. The induced and evoked responses are now considered separately.

#### 2.3.4. Evoked components

The source localization used whole-brain synthetic aperture magnetometry (SAM) [33,35]. The SAM images were generated by applying a multiple sphere forward model based on the participant's skull-stripped brain shape [36], which was derived from the previously acquired T1-weighted anatomical MR scan. Whole-brain covariance matrices were computed, with a 1–100 Hz bandpass filter, for each condition (‘Stop’ and ‘Go’) with the pre-stimulus baseline subtracted and the beam-former algorithm applied with a 4 mm spatial resolution [37], resulting in SAM images which were computed as voxel-wise pseudo t-statistics (e.g. [38]). The resulting ‘Go’ image was then subtracted from the ‘Stop’ image using FSLmaths (FMRIB, Oxford), and the active period, across which activity was analysed, was the participant-specific tROI, with the 500 ms baseline previously described. The peak activation from the resulting image was located within each aROI and a set of virtual sensors constructed based upon this.

The spatial restriction to the aROIs involved the use of a pair of binary masks, which were constructed using FSL and registered to native space. The pre-SMA mask was based on the Automated Anatomical Labeling atlas [39] where y is greater than 0 within the SMA region [1]. The IFC mask used the Harvard–Oxford atlas [40], consisting of the bilateral *pars triangularis* and the *pars opercularis*. Masks were thresholded to 25% [41]. The peak SAM activation, during the tROI, within each mask was then located for each participant using mri3Dx [42]. Virtual sensors were constructed on the basis of these derived coordinates (e.g. [43]), resulting in activity traces for both regions. Evoked virtual sensors were low-pass filtered to 100 Hz. Bidirectional zero-phase Butterworth filters were used for all frequency-specific filters.

These virtual sensors were used to produce evoked traces for each aROI. First, activity was averaged across trials for each trial class (‘Stop’ and ‘Go’) independently. The mean down-sampled ‘Go’ activity trace was then subtracted from the ‘Stop’ trace. Following this, the ‘fixation’ baseline, consisting of the final 500 ms before the arrow signal was presented [16], was subtracted. The fixation baseline period was, therefore, offset from the tROI by each participant's specific SSD plus 50 ms. The mean and variance over the fixation baseline was used to produce the evoked activity traces, expressed as a series of *z*-scores [44]. A 30 Hz low-pass filter was then applied [45]. The resulting activity traces combined information across trials, allowing latency quantification to be applied to activity related to response inhibition arising within each participant's aROIs.

**Figure 2.**
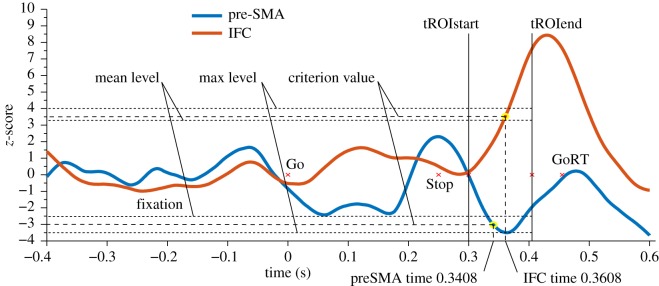
Example of a single participant's activity traces. The time courses of the events during trials are shown, including the end of the fixation period, the ‘Go’ and ‘Stop’ signal onsets, the participant-specific tROI and their mean Go reaction time (GoRT). The thicker dashed lines indicate the *z*-score criterion level, applied to each activity trace where the time point at which the trace crosses the criterion value is the latency-dependent measure. Also depicted are the mean and maximum level values used in the calculation of the criterion levels, where the absolute criteria levels were incremented in step sizes of 0.5 s.d. until the absolute mean was exceeded and the absolute maximum was at least 0.5 s.d. greater.

The latency of activity traces was calculated as the earliest time point during the tROI where activity deviated from baseline above a pre-specified criterion [46]. The criterion applied here was an adaptation of fractional latency quantification [47] which took into account both the mean fluctuation from baseline during the tROI and its peak (figure 2). Conventional fractional latency and peak latency quantification may have been inappropriate here as the tROI restriction led to incomplete coverage of full components and peaks [45]. The latency quantification involved identifying the time point where a criterion level was surpassed. The same criterion determination procedure was applied to each activity trace, but the resulting criterion level was derived for each participant's aROI activity trace independently. This reduced biases caused by, for example, gross amplitude differences in activity traces, independent of temporal progression, which can affect analyses where fixed criteria are applied. As activity traces were expressed as *z*-scores, the criteria were expressed in terms of standard deviations and were determined for each activity trace as follows: the initial criterion was set to 0.5 s.d. If both the mean activity during the tROI exceeded or was equal to this level, and the peak in activity was equal to or above this level plus 0.5 s.d., then the criteria were incremented by 0.5 s.d. This process was repeated until these conditions were no longer met. At this point latency was quantified as the first time point in each specific activity trace during the tROI where the *z*-score first surpassed the criterion level. This resulted in a single time point measure for each region, class of measures (evoked, *α*, *β* and *γ*) and participant.

If no fluctuation within the tROI of any particular participant's activity trace exceeded the most liberal criteria (mean *z* ≥ 0.5 s.d. and maximum *z* ≥ 1 s.d.), then that participant's data were excluded from the relevant group level comparison only (see §2.6). Alternatively, the conditions of the criteria may not be met in another respect, for example, where the peak in activity was very close (<0.5 s.d.) to mean levels. Were this applied, such a pattern was considered dominated by noise and as such the corresponding participant's data were excluded from the subsequent corresponding analysis. These exclusions were designed to ensure that only plausible physiological responses were entered into the analysis and are summarized in table 1.

**Table 1.** The number of participants excluded from each analysis on the basis of criteria applied to isolate only plausible physiological responses. Criterion 1 refers to the mean *z*-score of the activity trace during the tROI being ≥0.5 s.d.; criterion 2 refers to its maximum being ≥ 1 s.d.; and criterion 3 refers to its peak being within 0.5 s.d. of its mean.

	criterion						
measure	1 and 2 and 3	1 and 2	1 and 3	2 and 3	1	2	3
evoked	3	1	1	0	0	0	1
*α*	2	0	0	1	0	0	3
*β*	0	1	0	1	0	0	10
*γ*	0	0	0	2	0	0	9

The possibility of exploring multiple components was raised in the pre-registered protocol but was not pursued. This was due to the limited reliability of single components across the cohort (see §3.1), making the detection of multiple components unsound.

Following pre-registration and initial analysis, it became apparent that a contrast based on the difference between successful and failed stop trials was also capable of revealing cortical dynamics differences between the aROIs. It is possible that only when successfully stopping an action, one of the tROI's activities precedes the other. To probe this possibility, an additional exploratory analysis was therefore undertaken based on the contrast between successful and failed stop trials. Other than the replacement of the ‘Stop’ versus ‘Go’ contrast with one between ‘Successful Stop’ versus ‘Failed Stop’ trials, these analyses were identical except that no down-sampling was applied. This was because the threshold procedure resulted in roughly equal trial numbers across the contrast and trial numbers were already at, or below, an acceptable minimum (trial numbers: mean ± s.d. successful stop 63.42 ± 9.29; failed stop 52.68 ± 9.92).

#### 2.3.5. Induced components

The investigation of the induced responses focused on three broad-spectrum frequency pairings: *α* = 6 Hz to 13 Hz [16], *β* = 14 Hz [16] to 30 Hz [18,19] and *γ* = 31 Hz [16] to 130 Hz [4]. Following registration of the current protocol, response inhibition-related activity was also demonstrated in the lower *θ* range [6] and therefore may be an area for future investigation. Time-resolved oscillatory changes within these three frequency bands were quantified using the analytic signal, based on the Hilbert transform, to derive amplitude envelopes from the unbaselined datasets. These envelopes were derived at each frequency of the frequency pairings with a step size of 1 Hz and bandwidth of 8 Hz (e.g. [48]). The 1 Hz step size used deviates from the 0.5 Hz originally pre-registered, as 1 Hz was initially used to calculate the coordinates used in the application of the TMS. The progression of the experiments (TMS being applied based on measures constructed using the 1 Hz set size) meant it was not possible to reanalyse the data using a 0.5 Hz step size. Furthermore, a 0.5 Hz step size can be interpreted as inconsistent with the pre-registered frequency restriction procedures (see below). Initial source localization was carried out using a contrast between ‘Stop’ and down-sampled ‘Go’ trials, where the induced response was averaged across the tROI. The spatial restriction to the two aROIs then followed that described for the evoked responses, except the band filters made use of the frequency ranges described. Induced responses under the ‘Stop’ and ‘Go’ conditions were then subtracted from one another and the mean fixation baseline was subtracted and the activity trace was divided by the standard deviation of the baseline to produce induced time frequency spectrograms expressed as *z*-scores.

**Figure 3.**
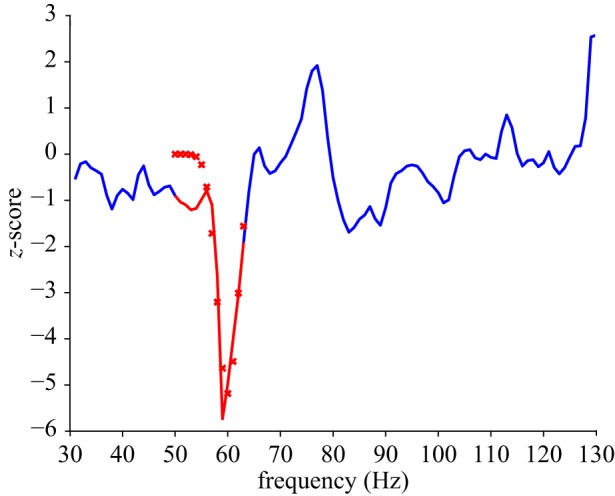
Example of the frequency restriction applied to single participant and aROI *γ* spectral data. Oscillatory change across a broad frequency range (e.g. 31–130 Hz) was collapsed across the tROI to produce a spectral response as depicted by the blue line. The red line indicates the frequency region resulting from a selection process (range selected is the maximum of a multiplication of the adjusted *R*^2^ values by the amplitude of the deflection). The fit is represented by the red crosses. The red region, therefore, represents the restricted frequency range used in subsequent analyses applied to this participant's aROI *γ* response.

Broad-spectrum band pairings (6–13 Hz, 14–30 Hz, 31–130 Hz) were used in the initial source localization steps. The *α* band in particular is broader than what is often considered *α* and therefore can alternatively be termed high *θ*. However, to avoid the inclusion of uninformative data (which may have been a particular problem within the *γ* range analyses), the resulting broadband spectra were further constrained in the frequency domain. This involved constructing multiple induced time frequency spectra, based upon permutations of potential band pairings within the initial broad range for each participant's dataset. The aim of the procedure was to obtain a frequency pairing that resulted in a veridical induced signal. The criteria used to determine which band pairing fulfilled this condition involved the application of a Gaussian model to the induced response collapsed across the tROI for both aROIs separately, for each participant's time frequency spectrograms for each of the three initial frequency ranges (*α*, *β* and *γ*, figure 3). Fitting was performed with Matlab in conjunction with the curve fitting toolbox, applying the Gaussian model:

$$y=a^{-{\left(\frac{x-b}{c}\right)}^{2}}.$$

Here *a* is the peak amplitude of the model, *b* is its frequency centroid position and *c* is the peak width across frequencies. The band pairing used was that which produced the highest value in a linear combination (multiplication) of adjusted *R*^2^ values from the Gaussian fit and the maximum absolute height of the deflection. An additional set of criteria was also applied within the frequency restriction to avoid local minima and reduce the likelihood of specifying single edges of the Gaussian models, as follows: for *α*, the minimum width of the pairings must contain at least 2 Hz, for *β* 4 Hz and *γ* 12 Hz. Additionally, pairings which resulted in a peak whose value was less than the values at the edges (i.e. a slope rather than Gaussian) were not to be used in preference to conventional Gaussian fits. This resulted in a single restricted band pairing for each participant and measures which should, theoretically, correspond to the frequencies where the greatest response to the stop task was expressed.

Once band restrictive criteria had been applied, the source localization process for each of the restricted frequency bands was then repeated, which resulted in time frequency spectra for each aROI, temporally restricted to each participant's frequency and tROI range. The spectral information was then collapsed across the band pairings to produce a single vector representative of the time course of oscillatory responses to a ‘Stop’ signal in the *α*, *β* and *γ* bands for both aROIs.

At this point the restricted and unrestricted traces were compared for each participant and frequency band. This involved the same criterion involved in the latency quantification (see §2.3.4) where the assumption was that a larger deflection from baseline (higher *z-*score) was indicative of a greater signal-to-noise ratio. Here, if the unrestricted traces resulted in a higher level of criteria being surpassed (e.g. *z* > 2.5 for the unrestricted trace, as opposed to *z* > 1.5 for the restricted trace), then the unrestricted induced traces were used for subsequent analyses. If the criterion level was equal across restricted and unrestricted applications, then the broader, unrestricted pairing was used. For these comparisons, between restricted and unrestricted traces, the mean criteria level obtained across the two aROIs was used. This procedure resulted in the mean band pairing summarized in table 2. The same method of latency quantification, as applied to the evoked responses, was then applied to the oscillatory induced traces, collapsed across the frequency range (figure 2).

**Table 2.** Mean and s.d. frequency band pairings used in the production of activity traces, for each induced measure across the group of participants. Restricted and unrestricted refers to the number of participants for whom the band pairing restriction (restricted) procedure was applied in the final analysis according to the pre-specified criteria.

	preSMA				IFC					
	mean		s.d.		mean		s.d.			
band	low	high	low	high	low	high	low	high	restricted	unrestricted
*α*	7.05	12.16	1.54	1.50	7.00	11.84	1.26	1.23	12	7
*β*	18.11	26.47	3.37	3.14	16.95	24.11	2.93	3.77	16	3
*γ*	70.26	92.84	29.85	31.91	71.05	90.89	30.52	30.47	18	1

#### 2.3.6. Granger causality analysis

An additional series of analyses explored the extent to which one time series (i.e. from one of the aROIs) was predictive of activity drawn from the other. Using Granger causality, we can test whether past activity from one region can predict activity in the other region to a greater extent than activity within a single region [22,23]. Although this analysis with the application of the toolbox provided by [23] was proposed in our pre-registered protocol, its details were not specified and therefore it can be considered as a secondary or exploratory set of analyses.

Our central question in these Granger analyses was whether activity derived from the pre-SMA aROI was predictive of activity from the IFC aROI, and/or vice versa. In particular, we asked whether past events in the pre-SMA reduce the prediction error of a linear regressive combination of the IFC and pre-SMA data to a greater extent than the IFC regressor alone, and whether this reduction is greater in magnitude than the reduction when past events, drawn from the IFC, are regressed upon the combination, over the pre-SMA alone [23].

Four aspects of activity were used for the comparisons between regions, as in the main analysis. Granger causality was applied to the raw virtual sensor activity trace during each participant's tROI, and in the frequency domain using the three initial band pairings (*α*6–13, *β*14–30, *γ*31–130 Hz) with a step size of 1 Hz via the application of a fast Fourier transform. The data were drawn from the previously described evoked virtual sensor which was recomputed using a 300 Hz low-pass filter and then made use of all non-excluded ‘Stop’ signal trials only. Data to which baselines were applied invariably resulted in persistent violations of the Granger assumptions of stationarity; therefore unbaselined data were used. The model orders were computed using the Bayesian information criterion [23] restricted to greater than four samples and less than the duration of the trial sample length minus four samples. This avoided interactions between the lag number and the filter properties. The data were de-trended and the temporal mean was removed using both the mean and s.d. Assumptions were checked using the augmented Dickey–Fuller test and where violations were found the data were repeatedly differenced until violations were eliminated (mean number of iterations (±s.d.) raw = 5.947(1.649), *α* = 5.684(1.529), *β* = 5.737(1.485), *γ* = 5.684(1.529)). This process was incapable of resolving violations to achieve covariance stationary data in one participant, who was thus excluded from these analyses. Following this, the model orders were recomputed as above, before the Granger regression was applied. The spectral analyses made use of randomization [23] at the sensor level (pre-SMA and IFC) to produce permutation tests using 500 surrogate data sets. At the group level (main statistics reported) the output causal density of the pre-SMA driving the IFC was compared with the causal density in the reverse direction with a series of paired *t*-tests and Bayesian equivalents using the Jeffreys–Zellner–Snow (JZS) prior (see §2.5). All complete MEG datasets were included in this analysis.

### 2.4. Transcranial magnetic stimulation

#### 2.4.1. Transcranial magnetic stimulation overview

We used functionally guided TMS in which single pulses of TMS were applied to both aROIs (pre-SMA and IFC) on every trial in one of two possible temporal orders (figure 1). If TMS applied to the pre-SMA before the IFC was more effective in impairing stopping ability than TMS applied in the opposite order, then this would imply a direction of information transfer from the pre-SMA to the IFC. Here we describe the procedure (§2.4.2), the equipment and parameters (§2.4.3) and the derivation of measures (§2.4.4). As with the MEG analyses, the primary analyses are described in a common statistical section (§2.5).

#### 2.4.2. Procedure

As TMS was functionally guided according to the MEG results, participants undertook the TMS session on a separate day, after their MEG session. In the TMS experiment, pairs of alternating sham and active TMS blocks consisting of 120 trials each were applied, the order of which (sham or active) was randomized for each participant. In the active condition, TMS was applied to both regions of interest; on half the trials the pre-SMA region was stimulated before the IFC and on the other half vice versa, with the order randomized for each block (see §2.4.3). In the sham condition, both coils were placed over approximately the same target locations but with the coil rim, rather than centre of the coil windings, placed over each target. This resulted in a sham coil orientation in which the coil was approximately at 90^o^ to that of the active condition, while allowing for the space constraints of the headrest.

Sham stimulation was used to baseline the data in order to control for the effects of confounds such as auditory distraction associated with coil discharge. The sham condition also served to maintain participants' performance around the optimal midpoint throughout the TMS experiment. Therefore, unlike in the MEG experiment, no performance feedback was provided to participants. If behavioural performance in the sham condition exceeded ±15% of the 50% successful stopping target, then the SSD was adjusted by the addition or subtraction of 50 ms from its onset (limited to a minimum SSD of 100 ms). The mean number of adjustments made to the SSD, across participants, was 0.833 out of a possible three opportunities. The resulting modification to the SSD was implemented for both active and sham trials during the subsequent pair of blocks. As the tROI, and therefore timing of the TMS, also depended upon the ‘Go’ reaction time (figure 1), the mean ‘Go’ reaction time and SSD during the previous sham block (or the mean Go RT during the previous MEG session, if conducted in the first experimental pair of blocks) were used to constrain the onset times of TMS. At the start of each experimental session, prior to the collection of experimental data, the participants completed a single block of 24 trials without TMS to re-familiarize themselves with the task. The data from this ‘pre’ block were not included in experimental analyses.

Over the course of the experiment, participants completed four active and four sham blocks; these were undertaken over one or two sessions depending upon participant availability. Overall this resulted in 480 active trials and 480 sham trials, with half of the trials in each case (240) assigned to each temporal TMS condition (IFG → pre-SMA, pre-SMA → IFG).

Motor thresholds were obtained during the calibration session using the observation of movement method [49,50]. The mean motor threshold (±s.d.) was 59.6% stimulator output (±7.2). The target TMS intensity applied during the experiment was 110**%** of the participant's distance-adjusted motor threshold. The distance adjustment, and corresponding intensity applied, depended upon the spatial locations of the MEG activations (§§2.3.4 and 2.3.5). For practical reasons, the threshold session preceded the MEG session; therefore approximations of the target locations were used during these sessions to establish participants' suitability for participation. This approximation involved calculating the Euclidean centre of the two aROI masks previously described (§2.3.4), where the IFC mask was restricted to the right hemisphere. The target locations were then used in the calculation of distance-adjusted stimulation intensity. This calculation required that an assumption was made about the site at which TMS was effective when motor responses were elicited. Here we assumed this point to be the Euclidean centre of a mask based upon the descriptions of the right motor hand knob provided by Yousry *et al*. [51] (also see [52,53]). This mask was constructed within the bounds of the Harvard–Oxford cortical atlas of the right pre-central gyrus and transformed into native space using FSLs FLIRT. Therefore, for each participant there were three target locations (pre-SMA, right IFC and the right motor hand area) used in the distance adjustment procedure. The closest scalp coordinate and the distance to these locations were then derived and the stimulation intensity adjusted to take account of differences in scalp–target distance. This calculation assumed that the rate at which TMS intensity drops off under such conditions is 2.7% per mm [54]. The scalp locations corresponding to the motor region were also used as the initial target location for the motor threshold. To prevent these adjustments resulting in intensities beyond what can reasonably be expected to have had an effect or be tolerated by the participant, an additional criterion was applied to each site, where TMS intensity was limited to 70–130% of each participant's MT. If the distance adjustment methodology returned intensities beyond this range, or above the maximum stimulator output, then boundary values were used.

During a calibration session and following the establishment of stimulation intensities for each region, a comfort threshold was conducted in which TMS was applied to both regions initially at 50% of the prescribed intensity. The intensity was then increased until the prescribed intensity was reached or the participant expressed discomfort. Manipulations in coil orientation were occasionally implemented if the prescribed intensity could not be obtained under initial conditions (§2.4.3). If the participant reported discomfort and this resulted in an adjustment of intensity to less than 80% of its original value, the participant was excluded (1 participant). If an adjustment was made to stimulation intensity due to the comfort threshold, or the maximum output was used over one region, then the TMS applied to the other region was adjusted by the same percentage of site-specific intensity. If the intensity applied to both regions was adjusted, then the greater of the two adjustments was used to specify the intensity at which TMS was applied to both regions. For example, if the intensity was initially prescribed to be 75% to the pre-SMA and 73% to the IFC, but the participant reported discomfort at these levels and was comfortable at only 73% for the pre-SMA and only 68% for the IFC, then TMS would have been applied at 68% to the IFC and at 70% to the pre-SMA, with the greater of the two reductions applied to the IFC. This resulted in the mean (±s.d.) TMS intensity to the pre-SMA of 73.6% stimulator output (±16.8%) and to the IFC of 69.2% stimulator output (±14.5%). This compares to the ideal intensity resulting from the distance adjustment procedure of 76.7% stimulator output (±18.2) for the pre-SMA and 71.9% (±16.2%) for the IFC. Reported as a percentage of the motor threshold, TMS was applied to the pre-SMA at 123.4% (±25.1) and to the IFC at 116.7% (±23.6).

#### 2.4.3. Transcranial magnetic stimulation equipment and parameters

Visual stimuli were presented using the same apparatus as described for the behavioural calibration (§§2.2.1–2.2.3). TMS was applied using two Magstim Rapid^2^ stimulators and two 50 mm figure-of-eight coils, targeted at both aROIs. The IFC coil was initially oriented over the IFC with the handle pointing upwards in the dorsal direction. The handle of the pre-SMA coil was pointed in a posterior direction (figure 1). Small adjustments to these orientations were made to improve comfort and due to spatial restrictions, while the centre of the coil windings was maintained over the target region.

The timing of the TMS pulses depended upon an individual participant's tROI. The first pulse was applied at the start of the closest screen refresh to one-eighth (12.5%) of the tROI and the second pulse was applied at the closest screen refresh to five-eighths (62.5%) of the tROI. These time points were chosen to balance the efficacy of pulses against independence of conditions, tailored to individual participant's performance.

Each virtual sensor used in the final set of MEG measures for each participant (see §§2.3.4 and 2.3.5) contributed to the definition of the TMS target site. As there were four MEG measures per participant per site, the Euclidean mean centre of these point locations was used. However, with respect to the IFC, the source localization process described above was capable of finding peak activation within either of the two hemispheres [6]. This left open the unsatisfactory possibility that any mean could lie between hemispheres. Under such conditions the mean was calculated within the hemisphere that shared a majority of the virtual sensors; for instance, if three out of four virtual sensors were located within the same hemisphere, then the mean of those three was used. Alternatively, if the virtual sensors were equally distributed across the hemispheres, then the right hemisphere's virtual sensor locations were used, as there is greater evidence for a role of the right IFC in stopping (e.g. [2,55]). This procedure resulted in 15 of the 18 participants receiving TMS to their right IFC. For the pre-SMA, the absolute Euclidean distance between the atlas-guided targets and the MEG-guided targets was 9.6(4.8) mm (mean (±s.d.)), and when applied to the scalp surface the distance was 10.9(4.8) mm. For the IFC, using only data where the MEG coordinates resolved to the right hemisphere, the Euclidean distance between targets was 18.7(8.5) mm and on the scalp surface it was 23.1(12.4) mm. The adjustment of stimulation intensity according to the scalp–cortex distance was the same as described for the calibration session with the exception that the TMS targets were now derived from the MEG data for the pre-SMA and IFC. If the prescribed intensity exceeded the restriction applied by the participant's comfort threshold, then the comfort level was used and an additional comfort threshold at the start of the first experimental session was also conducted, as described above.

TMS coils were positioned using a Brainsight system (Rogue Research Inc.) in conjunction with the participant's structural brain scan and were held over the anatomical target with tripod-mounted articulated arms (Manfrotto). Participants made use of a chin and forehead rest. Coil position, established at the start of each block, was maintained within a 5 mm tolerance of the desired scalp location. At the start of each block a test pulse was delivered through each TMS coil in order to ensure that the participant did not experience high levels of discomfort.

#### 2.4.4. Measures

The main dependent measure in the TMS experiment was the SSRT, calculated using the integration method [30,31]. SSRT reflects a chain of processes involved in stopping an ongoing action [56,57]. Active TMS was expected to disrupt the ability to stop an ongoing action relative to sham. The dependent measures were, therefore, baselined to the sham condition that had the same temporal order of TMS (active–sham, Δ). In addition to the primary SSRT analysis, secondary measures included the proportion of trials where stopping was successful, reaction times on ‘Go’ trials and failed ‘Stop’ reaction times.

### 2.5. Statistical analyses

#### 2.5.1. Difference tests

*T*-tests and complementary Bayesian statistics were used to ascertain if there was a reliable difference between the measures derived from the two sites. The *t*-test was two-tailed and paired. The principal Bayesian tests made use of the JZS prior described by Rouder *et al*. [58] with a default scaling factor of √0.5. Here a Bayes factor (BF) greater than three indicates substantial support for a difference between conditions, BF less than one-third provided substantial evidence that the two traces were indistinguishable and BF around 1 indicates that there is insufficient data to probe the question of temporal order [32].

In the MEG experiment, the primary comparison was between the latency measures drawn from each aROI, and in the TMS experiment it was between SSRT measures under different orders of active stimulation, baselined to sham. To investigate the specificity of TMS effects to response inhibition, an analysis of reaction time on ‘Go’ trials was also pre-registered. To further probe the functional specificity of observed effects of TMS, reaction time on failed stop trials and the proportion of successfully stopped trials were also considered in exploratory analyses. All analyses that were not pre-registered are labelled explicitly as exploratory.

Additional secondary Bayesian tests were implemented where priors were based upon previous research and theory. These are most appropriate for the MEG data but were additionally applied in exploration of the primary TMS measure, SSRT. The first of these was based upon the work of [59,60] which showed that direct connection between comparable regions may involve an approximately 30 ms offset. This finding can be summarized with a positive half normal prior applied to IFC minus pre-SMA data in which a single s.d. was equal to 30 ms starting at 0 ms [32]. Another alternative prior was based upon the consideration that it was unlikely that any temporal difference between data drawn from one region and the other would have been greater than half the duration of the tROI. Therefore, the variance of a half normal prior representative of a temporal difference between the two was set to half the group mean tROI duration [32]. The corresponding values were 71 ms in the TMS experiment and 63 ms in the MEG experiment.

The demonstration of reliable differences between sites, tested here, is central to the whole investigation. Because of this, the number of subjects participating in the whole experiment could have been determined according to Bayesian difference tests. As Bayesian hypothesis testing quantifies both the confidence that can be placed in the hypothesis, given the data, *and* its respective null, these tests do not suffer from the concerns of multiple comparisons and violation of stopping rules in frequentist statistics [32,61]. Therefore, the aim was to continue collecting data until both the following conditions were simultaneously met:

(i) The TMS experiment revealed substantial evidence that one of the temporal orders of TMS was more effective in suppressing stopping performance than the other (BF ≥ 3), or evidence indicated that the TMS protocol caused impairment of stopping performance (BF ≥ 3 active versus sham for both sites) that was indistinguishable according to their temporal order (BF < 1/3), as was the case with 20 participants (see §3.2)
(ii) The MEG experiment indicated that at least one of the derived measures contained evidence (BF ≥ 3) that activity drawn from one aROI preceded that from the other aROI. However, all of the MEG measures accumulated evidence that the temporal orders of events were indistinguishable between the two aROIs, with BF approaching 1/3 (see §3.1). This, together with practical considerations led to data collection being terminated at 20 participants.

#### 2.5.2. Correlation tests

The correlation analysis sought to examine potential relationships between the TMS and MEG data and the correspondence between the multiple MEG measures. This took the form of Pearson's correlations and Bayesian correlations [62], which again made use of the JZS prior. These were applied to the primary measures (latency of evoked, *α*, *β*, *γ* and SSRT active–sham) and subtracted data pertaining to the IFC (or IFC primacy in the case of the TMS measure) from that of the pre-SMA, to produce a single variable for each measure referring to any temporal difference between aROIs.

### 2.6. Exclusion criteria and participants

Below is a description of the pre-registered reasons why data could have been excluded from the analyses. All exclusions and reasons for exclusions are reported.

— Participants did not participate in the experiment if they did not pass the safety screening procedure for TMS approved by the ethics committee at Cardiff University, School of Psychology. The screening procedure was carried out at the start of the first calibration session.
— Participants were excluded from participation if their behavioural performance during the calibration session failed to meet the following conditions: if after six blocks of 120 trials they were unable to maintain 50% stopping performance when the SSD was equal to or greater than 100 ms, and reaction time on ‘Go’ trials was less than or equal to 550 ms. Participants may have also been excluded if they failed to follow task instructions.
— Following data collection, data were excluded from the corresponding experimental analyses only, if the participant failed to make ‘Go’ responses on more than 15% of ‘Go’ trials (no participants) or stopping performance was less than 30% or greater than 70% (one participant in the MEG experiment and one in the TMS experiment). For the TMS experiment, one point of clarification omitted in the original pre-registered protocol was that the criteria were applied to the sham control condition as their application to the active TMS condition had the potential to eliminate effects of interest.
— In the TMS experiment there had to be enough time between the onset of the ‘Stop’ signal and the mean ‘Go’ reaction time to allow for a difference between the temporal TMS conditions. That is, if the difference between their SSD and mean ‘Go’ reaction time was less than 117 ms, then, owing to the restriction of the tROI (figure 1), TMS would be applied under both TMS conditions at the same time. Therefore, any participant to whom this applied would have been excluded from the TMS experiment (no participants).
— If the participant reported discomfort during the application of the TMS and this required a reduction of TMS intensity to less than 80% of its original value, then the participant was excluded (two participants).
— MEG experimental data were excluded if excessive movement was detected (greater than 1 cm within a given block), or if the participant fell asleep during data collection. If more than 15% of trials were excluded at the visual inspection stage (see §2.3.2), then the participant's data were excluded. A participant's measure was removed from individual primary analyses if activity traces failed to reach the minimal criteria levels for inclusion (mean *z* > 0.5 and max *z* > 1) for either of the aROIs (see §§2.3.4 and 2.3.5).
— Participant's data may have been excluded due to other unanticipated technical failures (no participants).
— Participants may have withdrawn voluntarily from the study at any point (one participant).

### 2.7. Participant instructions

Participants were administered the following instructions prior to the experiment:

‘We are investigating how we control our actions. Specifically we are interested in how we stop an ongoing action. For this you have to make a response to stimuli, and then, occasionally, we will ask you to try to stop that action.

You will be shown white arrows. You should respond to these by pressing a button on the computer keyboard as quickly as possible. If you see the right arrow you should press the ‘K’ key, and if the arrow is pointing the left you should press ‘J’. Sometimes the arrows will turn black. This is your signal to STOP. That is, as soon as you notice the arrow has changed to black, try to not make the button press.

Do not wait for the black arrow. Just try to respond to the white arrow as quickly as possible on every trial and if the arrow turns black, then try your best to stop your response’.

## 3. Results

### 3.1. Magnetoencephalography results

The application of the behavioural criteria resulted in the exclusion of one participant whose stopping performance exceeded the pre-registered threshold of 70% across MEG experimental blocks. The behavioural response collected inside the MEG can be summarized, at the group level, as follows: the mean (±s.d.) SSRT was 210 (24) ms, the proportion stopped was 0.56 (0.09), SSD used was 202 (57) ms, reaction time on ‘Go’ trials was 428 (60) ms and the reaction time on failed ‘Stop’ trials was 386 (34) ms, which was shorter than the reaction time on ‘Go’ trials for all participants.

The criterion for inclusion into the evoked and induced analyses based upon the collection of discernible MEG responses was achieved by the following numbers of participants (evoked = 13, *α* = 13, *β* = 7, *γ* = 8), resulting in mean criterion *z*-score levels of (evoked = 1.269, *α* = 2.481, *β* = 1.821, *γ* = 1.594).

**Figure 4.**
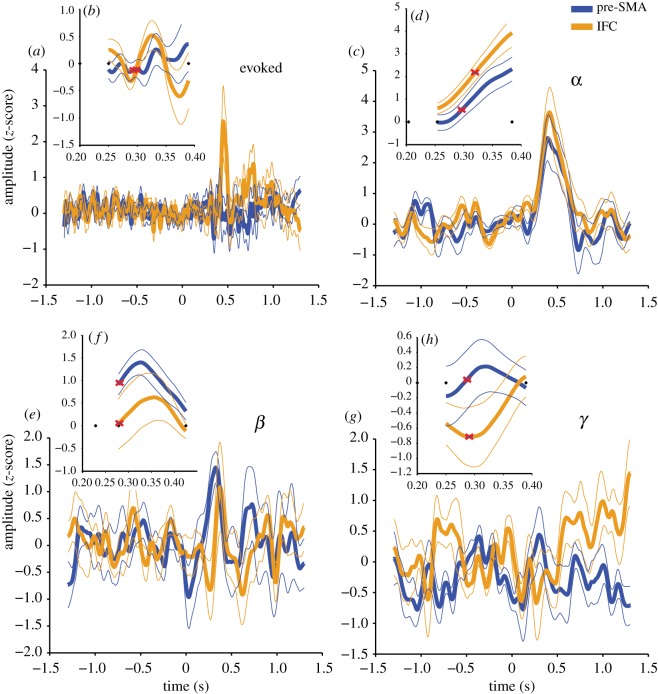
Group evoked (*a*,*b*) and induced *α* (*c*,*d*), *β* (*e*,*f*) and *γ* (*g*,*h*) activity traces during stopping. Main traces (*a*,*c*,*e*,*g*) are based on the (Stop–Go)–Fixation contrasts averaged across participants, following exclusions, from −1.3 to 1.3 s relative to the onset of the stimulus, and bandwidths applied in the induced traces are specified in §2.3.5. Insets (*b*,*d*,*f*,*h*) correspond to activity during the group tROI where individual participant data have been normalized to cover the group mean duration. Thick lines represent means across participants and thin lines are ±1 s.e. Red crosses indicate the mean point of deflection used in the temporal comparisons, for which the Bayesian comparison revealed concurrent activity across aROIs. Dots indicate the tROI from first onset of the stop signal, to the start and then the end of the critical tROI.

The onset of stop-signal dependent activity was indistinguishable between activity drawn from the pre-SMA and the IFC. The evoked traces exhibited appreciable deflections beyond those observed in the ‘Go’ condition (figure 4*a*,*b*) which were comparable in latency to the N2/P3 stopping-related components previously reported [12–14]. However, the pre-registered analyses demonstrated the onset of these responses to be comparable across anatomical sites (*T*_12_ = −0.857, *p* = 0.408, BF = 0.381). Likewise in the oscillatory domain, the *α* band analysis revealed induced responses (figure 4*c*,*d*) where the onset was demonstrably similar across sites (*T*_12_ = −0.717, *p* = 0.487, BF = 0.347). The *β* and *γ* traces also supported the null of there being equality in the onset of the induced response, although fewer participants’ data passed the criterion for the expression of a valid induced response (*β T*_6_ = −0.610, *p* = 0.565, BF = 0.411, *γ T*_7_ = −0.663, *p* = 0.529, BF = 0.403, figure 4*e*–*h*). Summary statistics and results of the application of alternative Bayesian priors are summarized in table 3. It is noteworthy that mean differences in the data, based on the pre-registered contrasts, were all in the direction of pre-SMA activity being followed by the IFC, which would be broadly consistent with previous findings [5,21]; however, no conclusion can be drawn with respect to the current data as evidence for such directionality appears to be insubstantial relative to the levels of random variation within the sample. The general form the deflections from baseline took, at the group level, were all increases in synchrony in oscillatory amplitude across the tROIs in both regions, and the evoked response exhibited an initial negative deflection followed by larger positive deflections.

**Table 3.** Summary of inferential pre-registered statistics applied to test differences in latency measures referring to the pre-SMA and the IFC. Negative *T* statistics indicate mean differences where the pre-SMA precedes the IFC. Bayes factors (BF) are reported using the primary default prior (JZS) and applying alternative priors. Of note, 30 ms refers to the 30 ms pre-SMA < IFC half normal prior and the 1/2tROI priors represent any difference as being most probably within half of the tROI (see §2.5).

	*T*	d.f.	*p*	JZS BF	30 ms BF	1/2tROI preSMA BF	1/2tROI IFC BF
evoked	−0.857	12	0.408	0.381	0.930	0.683	0.501
*α*	−0.717	12	0.487	0.347	0.833	0.543	0.409
*β*	−0.610	6	0.565	0.411	0.951	0.731	0.549
*γ*	−0.663	7	0.529	0.403	0.991	0.838	0.650

Table 4 summarizes the results of the exploratory unregistered temporal comparison but where data were contrasted between successful and failed stop signal trials. All related measures conformed to the outcomes of the pre-registered analyses, revealing invariance between the onset of pre-SMA and IFC activity. However, the results are more inconclusive which is likely to be due to the reduced signal to noise ratio caused by lower numbers of contributing trials.

**Table 4.** Summary of inferential statistics applied to test differences in latency measures for the pre-SMA and the IFC, based on the exploratory contrast between successful and failed stopping. All abbreviations are the same as in table 3.

	successful versus failed contrast						
	*T*	d.f.	*p*	JZS BF	30 ms BF	1/2tROI preSMA BF	1/2tROI IFC BF
evoked	−0.635	16	0.534	0.298	0.890	0.636	0.520
*α*	1.467	9	0.177	0.709	0.786	0.566	0.949
*β*	1.064	6	0.328	0.547	0.792	0.569	0.899
*γ*	−0.594	3	0.595	0.492	1.010	0.861	0.621

For completeness, it is possible to apply the same latency quantification as above but where the criteria for inclusion based on the deflections from baselines within the data (see §§2.3.4 and 2.6) have not been applied. These unregistered exploratory analyses again describe simultaneous latencies for the evoked (*T*_18_ = −0.625, *p* = 0.540, BF = 0.283), *β* (*T*_18_ = 0.040, *p* = 0.968, BF = 0.238) and *γ* (*T*_(18)_ = −0.332, *p* = 0.744, BF = 0.250) traces. There is a suggestion of a difference with respect to the *α* trace (*T*_18_ = −2.341, *p* = 0.031, BF = 2.067) in which the pre-SMA precedes the IFC, but this result should be treated with caution.

The exploratory Granger causality analysis applied to the raw virtual sensor data provided weak evidence in favour of primacy of the pre-SMA over the IFC (*T*_18_ = −2.415, *p* = 0.027, BF = 2.337). In the oscillatory domain, the mean Granger weights were higher in the pre-SMA to IFC direction, possibly suggesting primacy of the pre-SMA in all three frequency bands. However, these trends were statistically insubstantial, with moderate support for the null in the case of the *β* band (*α T*_18_ = 1.814, *p* = 0.086, BF = 0.931; *β T*_18_ = 0.471, *p* = 0.643, BF = 0.262; *γ T*_18_ = 1.138, *p* = 0.270, BF = 0.418).

### 3.2. Transcranial magnetic stimulation results

Two participants withdrew from the TMS experiment due to discomfort and one was excluded on the basis of excessive stopping (>70% as pre-registered). Group mean behavioural performance, following exclusions, across TMS conditions was as follows (mean (±s.d.)): SSRT: 239 (24) ms, reaction time on ‘Go’ trials 419 (54) ms, on failed ‘Stop’ trials it was 375 (35) ms, proportion stopped 0.431 (0.078) and failed ‘Stop’ reaction time was shorter than ‘Go’ reaction time for all participants. The group mean SSD used was 179 (59) ms. In the sham condition alone, the mean SSRT was 231(27) ms, reaction time on ‘Go’ trials 416 (52) ms; on failed ‘Stop’ trials it was 370 (36) ms and the proportion stopped was 0.46 (0.09).

**Figure 5.**
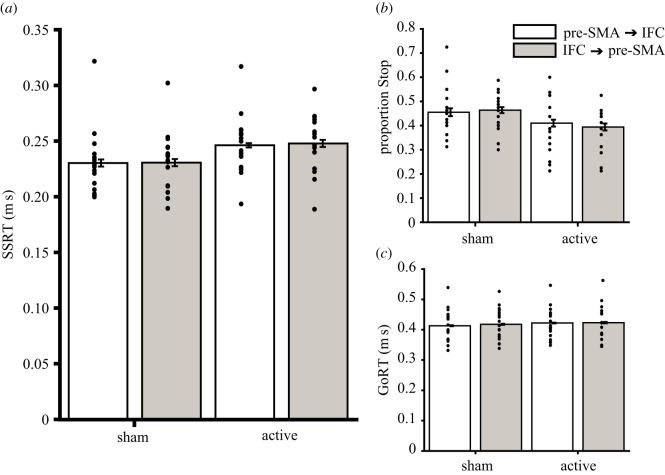
Behavioural effects of pre-SMA and IFC stimulation on response inhibition and response execution. Bars indicate group mean SSRT(*a*), proportion stopped (*b*) and ‘Go’ RT (*c*) under each of the TMS conditions (sham and active, as well as where TMS is applied to the pre-SMA before the IFC and where TMS is applied to the IFC before the pre-SMA). Individual data points are shown to the left of within-participant standard error bars [63].

The primary pre-registered comparison of the change in SSRT from sham between the two temporal orders of stimulation (IFG then pre-SMA versus pre-SMA then IFC) revealed no evidence of a difference, with substantial support for the null hypothesis of no difference (*Δ*SSRT: *T*_16_ = −0.276, *p* = 0.786, BF = 0.258, figure 5). However, secondary analyses indicated that the TMS intervention was generally effective in impairing stopping performance, with SSRT significantly elevated during active TMS relative to sham (*T*_16_ = 3.825, *p* = 0.002, BF = 26.77). This, in turn, was accompanied by a reduction in the proportion of successfully stopped trials (active versus sham, *T*_16_ = 2.522, *p* = 0.023, BF = 2.757) as opposed to non-specific effects upon ‘Go’ reaction time (active versus sham, *T*_16_ = 1.605, *p* = 0.128, BF = 0.727). This increase in the SSRT was present under both temporal order conditions (initial pre-SMA TMS active versus sham *T*_16_ = 3.754, *p* = 0.002, BF = 23.544, initial IFC TMS active versus sham *T*_16_ = 3.082, *p* = 0.007, BF = 7.110). There was no evidence that the temporal order in which TMS was applied caused changes in secondary measures (Δ Go RT *T*_16_ = 0.916, *p* = 0.373, BF = 0.359, Δ proportion stopped *T*_16_ = 1.409, *p* = 0.178, BF = 0.576, *Δ* failed stop RT *T*_16_ = 0.658, *p* = 0.520, BF = 0.302).

A number of alternative informed priors and corresponding Bayesian analyses of the primary SSRT measure were pre-registered. These support the finding of invariance between TMS conditions, as was suggested with the use of the default prior. When the prior was based on the 30 ms precedence of the pre-SMA over the IFC, the data supported the null hypothesis, but not conclusively (BF = 0.532). When a uniform prior was constructed based upon the group level tROI, substantial support for the null hypothesis was observed irrespective of the which region was primary in the prior's construction (IFC- > pre-SMA BF:0.292, pre-SMA- > IFC BF:0.263).

To what extent was the observed effect of TMS specific for SSRT compared with the other dependent measures? To answer this question, we performed an additional exploratory analysis to compare the effects of TMS directly between behavioural measures, normalizing each measure to the % change from sham to ensure comparability of effects with baseline. We found that the % increase in the SSRT in active relative to sham conditions (mean = +7.6%, s.e. = 2.0%) was significantly greater than the % change from sham in GoRT (mean = +1.8%, s.e. = 1.1%, *T*_16_ = 3.058, *p* = 0.007, BF = 6.82; substantial evidence for H1) and the % change from sham for failed response RTs (mean = +2.5%, s.e. = 3.9%, T_16_ = 2.369, *p* = 0.031, BF = 2.157; anecdotal evidence for H1), while GoRTs and failed response RTs did not differ significantly from each other (*T*_16_ = 0.788, *p* = 0.442, BF = 0.327; substantial evidence for H0). These results provide evidence that the impairment in behaviour during active versus sham TMS was relatively specific for SSRT.

### 3.3. Correlation analyses

These pre-registered analyses tested potential relationships between measures which may have indicated common underlying processes. One of these suggested significant (*p* < 0.05) relationships between the latency of the *γ* and *β* responses (table 5). However, the corresponding Bayesian [62] analyses suggested that the evidence was inconclusive and, furthermore, the significance of this frequentist analysis was eliminated when corrections for multiple comparisons were applied (here *p* ≤ 0.005 [64]).

**Table 5.** Summary of correlation analysis designed to quantify relationships between the primary measures of interest. The measure used to quantify the TMS effect was the SSRT (active–sham) and for MEG data the latency measures are used for each evoked (evo) and induced change in each band.

	*R*					d.f.					*p*					BF			
	evo	*α*	*β*	*γ*		evo	*α*	*β*	*γ*		evo	*α*	*β*	*γ*		evo	*α*	*β*	*γ*
TMS	−0.116	−0.117	−0.267	0.072	TMS	15	15	15	15	TMS	0.658	0.655	0.301	0.784	TMS	0.203	0.203	0.313	0.191
evo		−0.011	0.136	−0.419	evo		17	17	17	evo		0.965	0.580	0.074	evo		0.175	0.204	0.854
*α*			0.203	−0.154	*α*			17	17	*α*			0.405	0.529	*α*			0.247	0.213
*β*				−0.493	*β*				17	*β*				0.032	*β*				1.711

## 4. Discussion

This study examined the temporal primacy of the pre-SMA and the IFC in response inhibition. Although exploratory analyses provided weak evidence consistent with a drive of activity from the pre-SMA to the IFC, overall the primary outcomes suggested that the functional time course of response inhibition was indistinguishable between the two regions. While we cannot rule out the possibility of a mediating third region, we believe the most likely interpretation of the invariance in the data, drawn from the two aROIs, is that it results from the parallel nature of processing and interplay between the two structures [64–66]. That is, a series of mutually interdependent recurrent interactions may form the basis of stopping a response. This was perhaps most clearly exhibited in the TMS experiment where application of TMS to both regions disrupted stopping, consistent with the critical role of both the pre-SMA and IFC in inhibitory control [2,3]. This represents a relatively specific perturbation of the stopping process as action execution was comparatively unaffected by the intervention. Importantly, however, the temporal order in which TMS was applied to the regions made no difference to the disruption of stopping, as might be expected when interfering with a densely interconnected mutually interdependent parallel process in which simultaneous activity within and between both regions is required to perform an act of voluntary inhibitory control [65,67]. This causal evidence contrasts with previous suggestions, based on correlational evidence, of temporal primacy of one region over the other (e.g. [4]).

Parallel, temporally simultaneous dynamics are also supported by the MEG data, where the Bayesian analysis in particular indicated that both regions expressed appreciable activity changes during response inhibition, but that these changes happened at approximately the same point in time. The approach we used to temporally quantify MEG responses appeared to successfully delineate the time points at which each region became active during response inhibition, with data excluded from the primary analysis where successful delineation of a response could not be determined. Also, the time points derived were consistent with previous research (e.g. [68,69]) and theory, situated within the first half of the tROI to enable sufficient time for a response inhibition-related signal to have reached the aROIs and sufficient time for either region to have exerted influence on motor action. We, therefore, interpret the data as suggesting that the time course of activity originating from the two regions during response inhibition is roughly simultaneous, possibly mirroring high levels of mutually interdependent activity. These findings were based on pre-registered analyses and were complemented by an exploratory contrast of successful versus failed stop trials, which indicated a similar parallel activity. Together these results can be interpreted as evidence against the idea that temporal primacy of IFG or pre-SMA is pivotal in response inhibition. Instead, successful response inhibition may be governed by the integration of ‘Go’ and ‘Stop’-related activity, possibly further downstream within basal ganglia and thalamic structures [1].

The application of Granger causality to the MEG data did provide weak evidence that activity within the pre-SMA may drive activity within the IFC, which is consistent with previous research [5,6,21,70]. However, this finding is exploratory (unlike the analyses of evoked and the three oscillatory responses) and was only present in the raw activity trace and, therefore, may not survive correction for multiple comparisons if the oscillatory comparisons are all deemed equally capable of informing the hypothesis. Furthermore, Granger causality tests a different question from that of the main pre-registered analyses; it quantified the predictive relationships during the whole tROI, as opposed to analysing the timing of a discernible event. This means that while neither region has been shown to initiate or precipitate a cascade of events in the response inhibition process, it is possible that there is an ongoing drive from pre-SMA to IFC during the interplay between the regions during stopping, as suggested by the Granger analysis. Previous research has suggested that this drive may be more generalized, encompassing periods beyond and preceding the initiation of a ‘Stop’ or even a ‘Go’ command [5,6]. Hence, consistent with the current interpretation, a pre-SMA to IFC drive may correspond to a proactive state of preparation, as opposed to a specific direction of influence in the actual stopping of an action.

The response inhibition literature generally lacks studies probing the question of temporal dynamics between cortical structures in response control, with a few notable exceptions (e.g. [21]). One recent study [6], published after the pre-registration of this study, examined the interplay between the pre-SMA and the IFC using MEG. Although an informative, complex and context-dependent relationship between the two regions was uncovered, the authors concluded that pre-SMA activity preceded that of the IFC. However, this was based upon a series of observations, including a *γ* band response, originating from the pre-SMA before the presentation of the signal to inhibit actions. Therefore, as previously suggested [56,71–74], and consistent with the current interpretation, the pre-SMA may be pivotal to the proactive state of inhibition preparation.

In conclusion, the current results suggest that activity within the pre-SMA and the IFC during the inhibition of an action may be effectively simultaneous. This implies that the basic question of temporal primacy, particularly in the form of which region is active or critical first, may fail to capture the complex interplay of cortical dynamics that occur during the cessation of actions.

## Data accessibility

The data and analysis materials, together with the original study pre-registration for this experiment are hosted on the Open Science Framework. The main project page is https://osf.io/rfxhn/ and the study pre-registration is available at https://osf.io/u4vs5/. The data can be downloaded at https://osf.io/qj66a/. The repository also contains a document summarizing the data and analysis software provided (StopMEGTMS_summary_open_data.docx). All data and analysis software have been provided, with the exception of data that could jeopardize anonymity of participants or analysis software for which we do not own the intellectual property rights.
